# Preservation of Antiviral Immunologic Efficacy Without Alloimmunity After Switch to Belatacept in Calcineurin Inhibitor–Intolerant Patients

**DOI:** 10.1016/j.ekir.2022.10.015

**Published:** 2022-10-20

**Authors:** Joanna Schaenman, Maura Rossetti, Harry Pickering, Gemalene Sunga, Holly Wilhalme, David Elashoff, Qiuheng Zhang, Michelle Hickey, Uttam Reddy, Gabriel Danovitch, Elaine F. Reed, Suphamai Bunnapradist

**Affiliations:** 1 Division of Infectious Disease, Department of Medicine, David Geffen School of Medicine, University of California Los Angeles, Los Angeles, California, USA; 2 Department of Pathology and Laboratory Medicine, David Geffen School of Medicine, University of California Los Angeles, Los Angeles, California, USA; 3 Department of Medicine Biostatistics Core, David Geffen School of Medicine, University of California Los Angeles, Los Angeles, California, USA; 4 Division of Kidney Transplantation, David Geffen School of Medicine, University of California Los Angeles, Los Angeles, California, USA

**Keywords:** belatacept, CMV, immunosuppression, transplantation, T cell

## Abstract

**Introduction:**

Belatacept has shown potential for prevention of rejection after kidney transplantation, given its demonstration of reduced nephrotoxicity in combination with absence of significant incidence of rejection. However, concerns have been raised regarding increased risk of viral infection.

**Methods:**

We set out to explore the impact of the switch to belatacept on alloimmune and antiviral immunity through the study of patients switched from calcineurin inhibitor (CNI) to belatacept within 3 months of kidney transplantation compared with a matched cohort of control patients on a CNI-based regimen.

**Results:**

After the switch to belatacept, immune phenotyping demonstrated a decrease in naive and an increase in terminally differentiated effector memory (TMRA) T cells, with no significant difference compared with control patients. Donor-specific immune response, measured by intracellular cytokine staining (ICS), did not change significantly either by single or double cytokine secretion, but it was associated with the appearance of donor-specific antibody (DSA) in the control but not the belatacept cohort (*P* = 0.039 for naive and *P* = 0.002 for TMRA subtypes). Increased incidence of *de novo* DSA development was observed in the control group (*P* = 0.035). Virus-specific immune response, as measured by ICS in response to cytomegalovirus (CMV) or Epstein-Barr virus (EBV), was similar in both groups and stable over time.

**Conclusion:**

We found that belatacept use was associated with an absence of alloreactivity without impact on immune phenotype, while preserving the antiviral immune response, for patients switched from a CNI-based regimen. In parallel, the antiviral immune response against CMV and EBV was preserved after the belatacept switch (clinicaltrials.gov: NCT01953120).

## Graphical abstract

**Figure:**
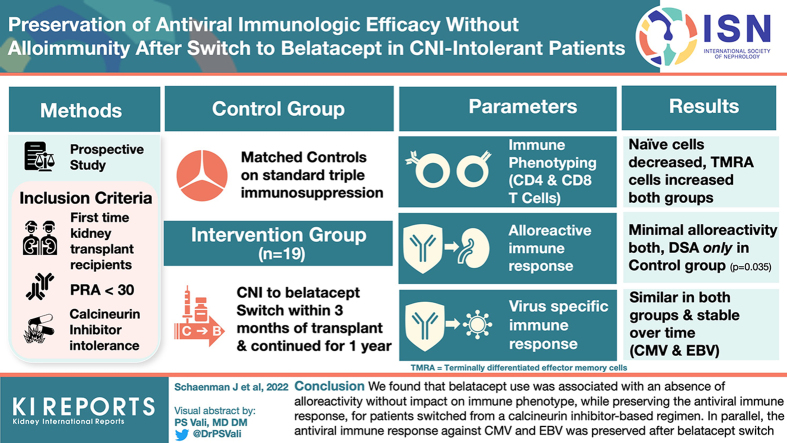

Costimulation blockade is a new approach for maintenance immunosuppression with increasing data on the safety and utility of this approach, including avoidance of CNI-associated toxicities.^1^, ^2^, ^3^ Belatacept is the most commonly used costimulation blocker as either initial immunosuppressive therapy or with switch in the setting of CNI intolerance.^4^, ^5^, ^6^, ^7^, ^8^ However, despite these promising results in terms of DSA and rejection, uptake in the transplant community remains low with only 3% of patients in the United States receiving *de novo* belatacept regimens.^9^

Belatacept was engineered as a costimulation pathway inhibitor, inhibiting T-cell maturation and activation by binding the ligands CD80 and CD86 to prevent binding to CD28.^10^ Given the observation that patients treated with belatacept may have increased rates of acute rejection despite evidence of superior long-term renal function,^11^, ^12^, ^13^ it is important to understand how switch to belatacept may affect the allo-antigen immune response. An increased frequency of posttransplant lymphoproliferative disorder in patients naive for EBV with EBV-positive donors (D+/R−) was also seen,^12^ although this association was not confirmed in a meta-analysis.^14^ Risk for EBV-associated disease may reflect inhibition of the development of antiviral immunity, but it is unclear how important the presence or lack of virus-specific memory T cells at the time of transplant may be. In addition, increased rates of CMV DNAemia and disease and impaired development of the anti-CMV cellular immune response have been reported.^15^, ^16^, ^17^, ^18^ There is a lack of previous *ex vivo* data in human subjects receiving belatacept comparing in parallel the impact of belatacept treatment on immune phenotype, alloimmune, and antiviral immune response using a flow cytometry–based approach, with previous data limited to exogenous addition of belatacept *in vitro*.^19^^,^^20^ Therefore, it is important to understand how CMV-specific T-cell immunity may be affected in the context of kidney transplant after belatacept switch from CNI therapy. In this cohort of patients switched to belatacept compared with a matched cohort of patients on conventional therapy, we have the ability to answer this question.

## Methods

### Clinical Care

In a prospective study to evaluate the immunologic impact of switch to belatacept immunosuppression (NCT01953120), 19 patients with evidence of CNI intolerance were enrolled and switched from CNI to belatacept within 3 months of transplantation. All patients signed informed consent. The University of California, Los Angeles Institutional Review Board approved this study. Study patients received intravenous belatacept at 5 mg/kg every 2 weeks at day 1 and weeks 2, 4, 6, and 8 and then monthly at months 3, 4, and 5. At month 6, patients were allowed to elect to continue for an additional 6-month period of belatacept administration. Definition of CNI intolerance was defined as neurologic toxicity, renal toxicity (glomerular filtration rate <60), metabolic toxicity, or hematologic toxicity. Inclusion criteria included first-time kidney transplant recipients with panel-reactive antibody <30% at the time of transplant. CNI therapy was tapered over 30 days. Minimum mycophenolate mofetil dosing was 500 mg by mouth twice daily, with prednisone at 10 mg daily at study entry if <6 weeks after transplantation, tapered to 5 mg daily, or prednisone 5 mg daily if >6 weeks after transplantation. Blood samples were collected at baseline, after transplantation but before starting belatacept, and then at 1, 3, 6, and 12 months after the switch to belatacept.

Patients were matched based by age, induction (antithymocyte globulin [ATG] vs. basiliximab), and donor type with 19 control patients with biobanked samples available, previously enrolled in an observational study of kidney transplant recipients at our center, with blood samples available at comparable time points compared with study patients. Baseline sample was defined as the first sample available at the same relative time after transplant compared with their matched study patient. Matching was performed before review of posttransplant outcomes. Given the pilot nature of this study, no formal power calculation was performed, and study accrual was based on subject and sample availability.

Peripheral blood mononuclear cells (PBMCs) were isolated and frozen for storage as previously reported; our previous studies demonstrate that this process does not significantly affect cell viability and ability to measure antigen-specific immune response.^21^^,^^22^ Details of immunosuppression and antibiotic prophylaxis were as previously described.^21^ Control patients received similar maintenance immunosuppression regimens with protocolized target drug levels and monitoring for infection. In brief, prophylaxis consisted of valganciclovir 900 mg by mouth daily for 6 months if the donor was positive and the recipient was negative for CMV by antibody testing. Recipients with positive CMV antibody received valganciclovir 900 mg by mouth daily for 3 months if they underwent ATG induction. Recipients with positive CMV antibody who received basiliximab for induction, or for whom both donor and recipient were CMV seronegative, received 3 months of antiviral prophylaxis with acyclovir 400 mg by mouth twice daily. Patients were characterized as having rejection during the first year after transplantation based on chart review using standard clinical criteria as previously reported.

### Immunologic Assessment

Immune phenotype was analyzed by multiparameter flow cytometry. Fixable Aqua Dead Cell Stain (Invitrogen, Waltham, MA) was used to identify intact and alive lymphocytes (Supplementary Figure S1). T-cell maturation was assessed using fluorochrome-conjugated antibodies against CD3, CD4, CD8, CCR7, and CD45RA to determine maturation phenotype. Naive cells were defined as CCR7+/CD45RA+, central memory as CCR7+/CD45RA−, effector memory as CCR7−/CD45RA−, and terminally differentiated as CCR7−/CD45RA+ (antibodies obtained from BD Biosciences or Biolegend). Exhaustion, senescence, and activation of T cells was evaluated using KLRG1, CD57, CD38, CD28, and PD-1. T-regulatory cells (Tregs) were defined as CD4+ CD25+CD127−. Lymphocyte populations were identified based on light scatter parameters, followed by live/dead gating and further gating on CD3+ expression. Detection was performed using the BD LSR Fortessa (BD) flow cytometer with data analysis by FlowJo software. Percentage of each relevant cell type is presented with reference to the denominator of CD4 or CD8 T cells.

Antigen response was determined by ICS for cytokines using donor cells or overlapping peptides representing the most immunodominant CMV or EBV antigens using previously reported techniques with demonstrated association between immune response, CMV serostatus, and protection from viremia (UL55, UL83 or pp65, UL99, UL36, UL48_sub1, UL48_sub2, UL122 or IE-1, UL123, and US32 (5 μg/ml, JPT Peptide Technologies, Berlin, Germany).^22^^,^^23^ Mixed lymphocyte reaction was performed using irradiated donor antigen presenting cells (10^6^) resuspended in 0.5-ml 10% human AB serum in RPMI medium and incubated with recipient PBMCs for 15 hours, as previously described.^24^ This method has been shown in previous studies to correlate with rejection risk.^25^ Third-party donor cells were used as negative control for alloimmunity assessment. Golgi plug and costimulatory signal (CD28/49d) (BD Biosciences) were added during stimulation. Positive control was incubation with Staphylococcus enterotoxin B (Sigma). Negative control was performed with absence of antigen-specific stimulation, incubating in otherwise identical conditions. Thawed PBMCs were incubated with either irradiated donor cells or overlapping peptide pools representing immunodominant CMV or EBV antigens overnight. Cells were stained for surface markers and then fixed and permeabilized for ICS. Live cells were identified, and flow cytometry performed as described above. Background staining was subtracted from each stimuli before analysis. Patients for whom neither donor nor recipient was positive for CMV were excluded from CMV-specific antigen testing. The percentage of each single or double cytokine-secreting cell type is in reference to the denominator of CD4 or CD8 T cells. For maturation analysis, the denominator was the total percentage of interferon-γ (IFN-γ/tumor necrosis factor-α (TNFα)–expressing CD8 or CD4 T cells, as appropriate. Single-antigen human leukocyte antigen class I and II testing was performed at each study time point; positive DFA was defined as any mean fluorescence intensity over 1000 detected after transplantation.

### Statistical Analysis

**Figure 1:**
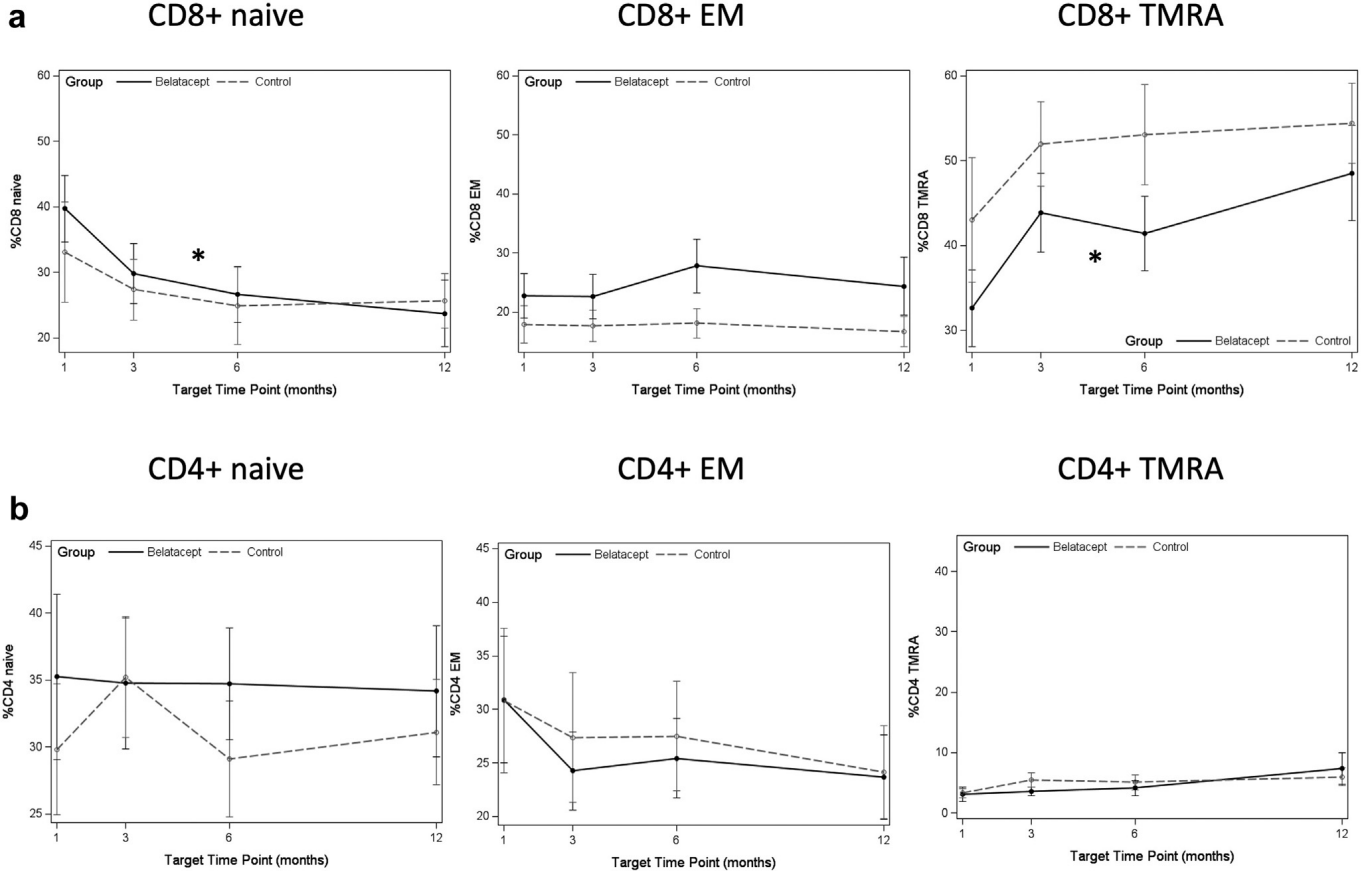

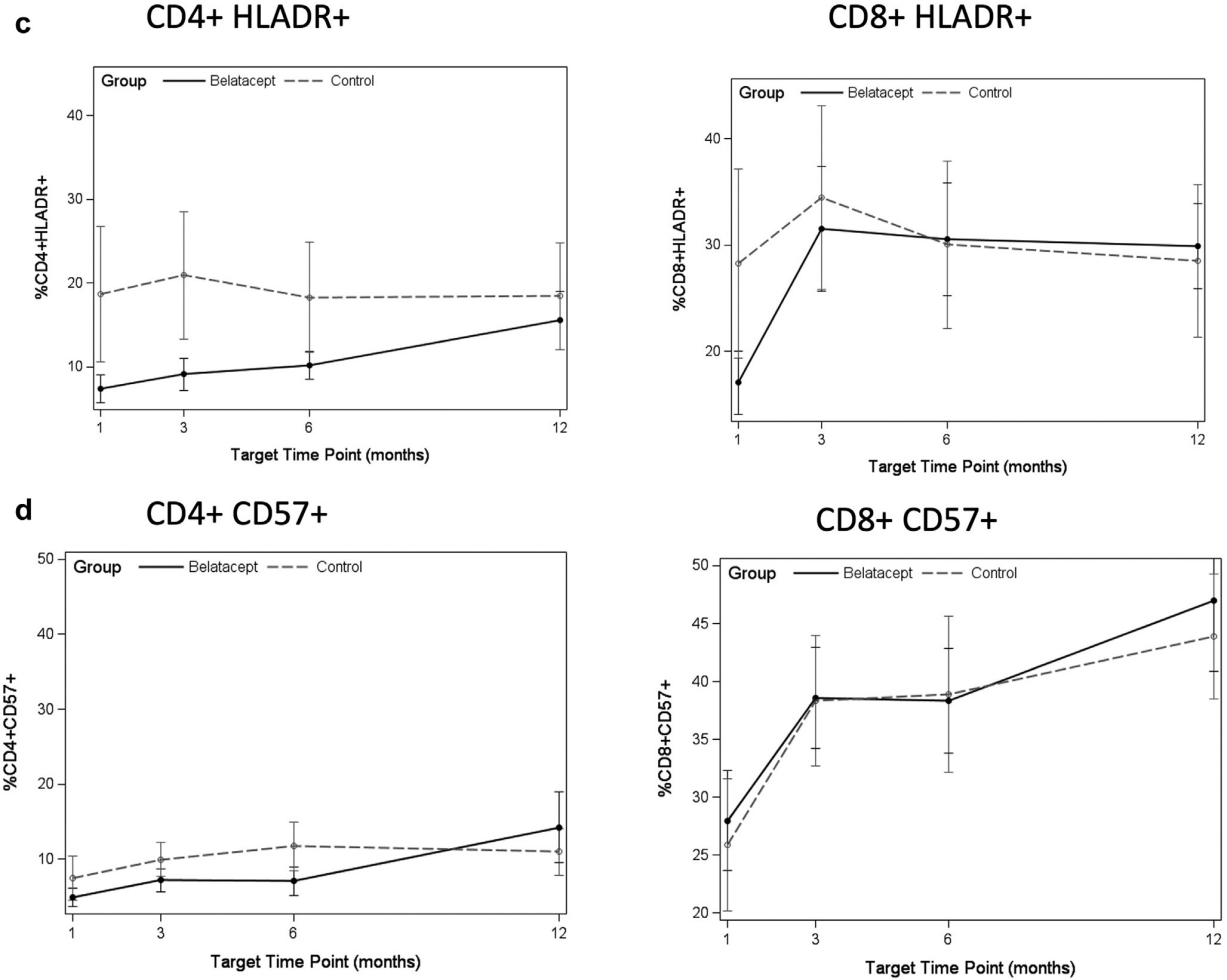
(a) Median frequency of CD8+ T-cell maturation subtypes at each time point after switch to belatacept, or equivalent time post-transplanted for controls. (b) Median frequency of CD4+ T-cell maturation subtypes at each time point after switch to belatacept, or equivalent time post-transplanted for controls. (c) Median frequency of activated T cells by HLADR+ at each time point after switch to belatacept, or equivalent time post-transplanted for controls. (d) Median frequency of activated T cells by CD57+ at each time point after switch to belatacept, or equivalent time post-transplanted for controls. Belatacept patients indicated by solid points and solid lines; control patients indicated by gray points and dashed lines. Asterisk indicates statistically significant change in slope (*P* < 0.05) by FDR testing measured by mixed-effect analysis. Denominator is the total percentage of CD8 or CD4 T cells, as appropriate. EM, effector memory; HLADR, X; TMRA, terminally differentiated effector memory cell.

**Figure 2:**
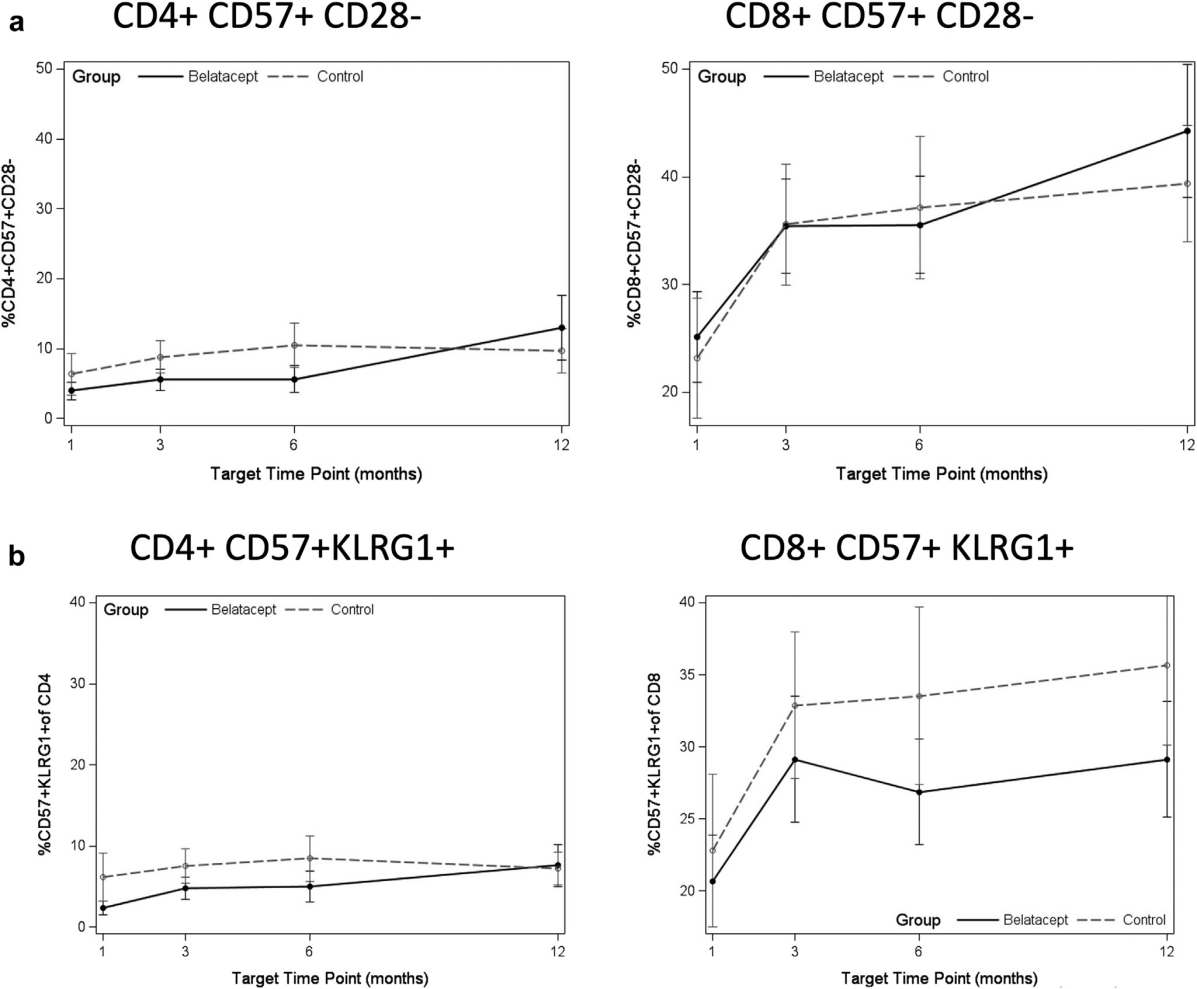

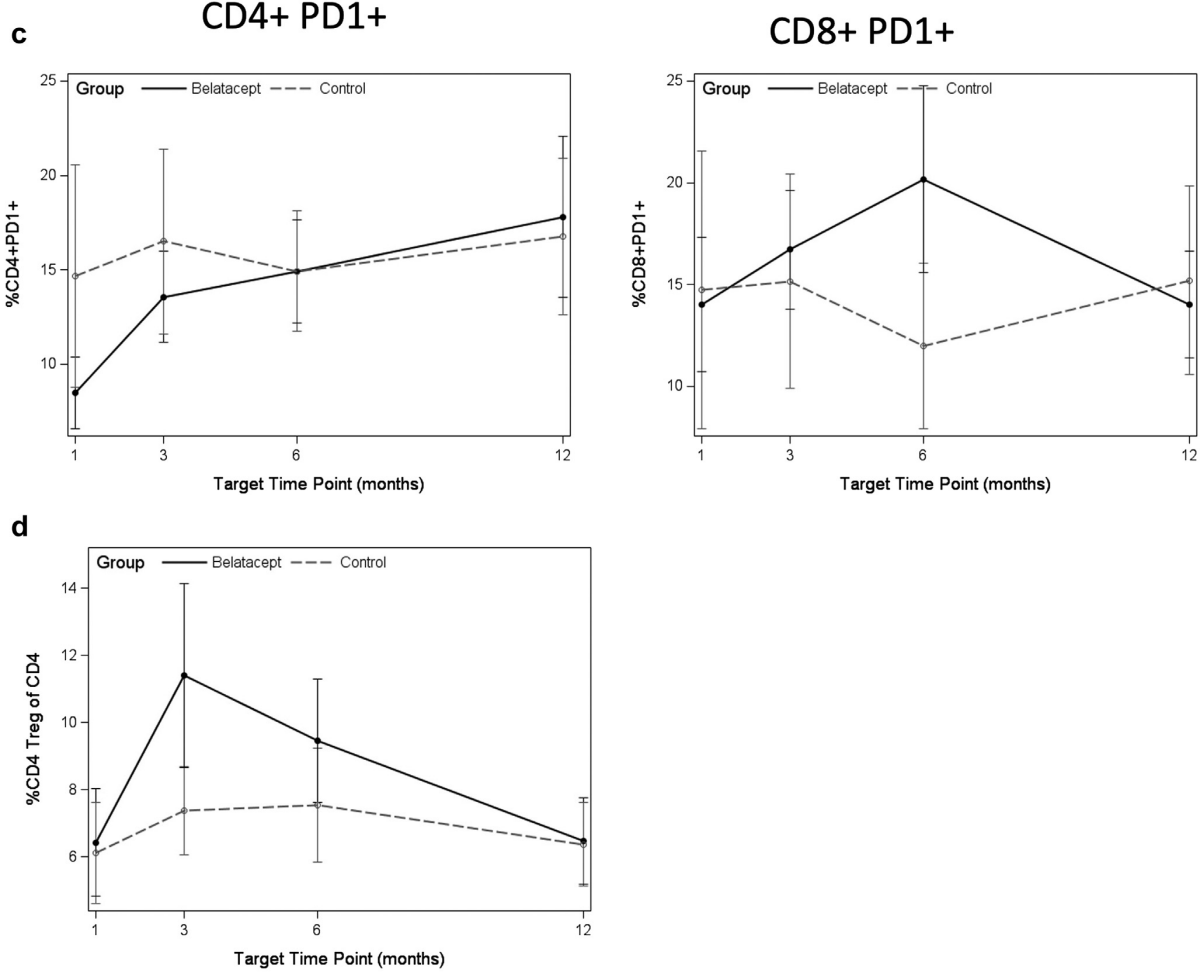
(a) Median frequency of CD57+CD28− senescent T cells at each time point after switch to belatacept, or equivalent time post-transplanted for controls. (b) Median frequency of CD57+KLRG1+ senescent T cells at each time point after switch to belatacept, or equivalent time post-transplanted for controls. (c) Median frequency of PD1+ exhausted T cells at each time point after switch to belatacept, or equivalent time post-transplanted for controls. (d) Median frequency of CD4+ Tregs at each time point after switch to belatacept, or equivalent time post-transplant for controls. Belatacept patients indicated by solid points and solid lines; control patients indicated by gray points and dashed lines. Asterisk indicates statistically significant change in slope (*P* < 0.05) by FDR testing. Denominator is the total percentage of CD8 or CD4 T cells, as appropriate.

**Figure 3:**
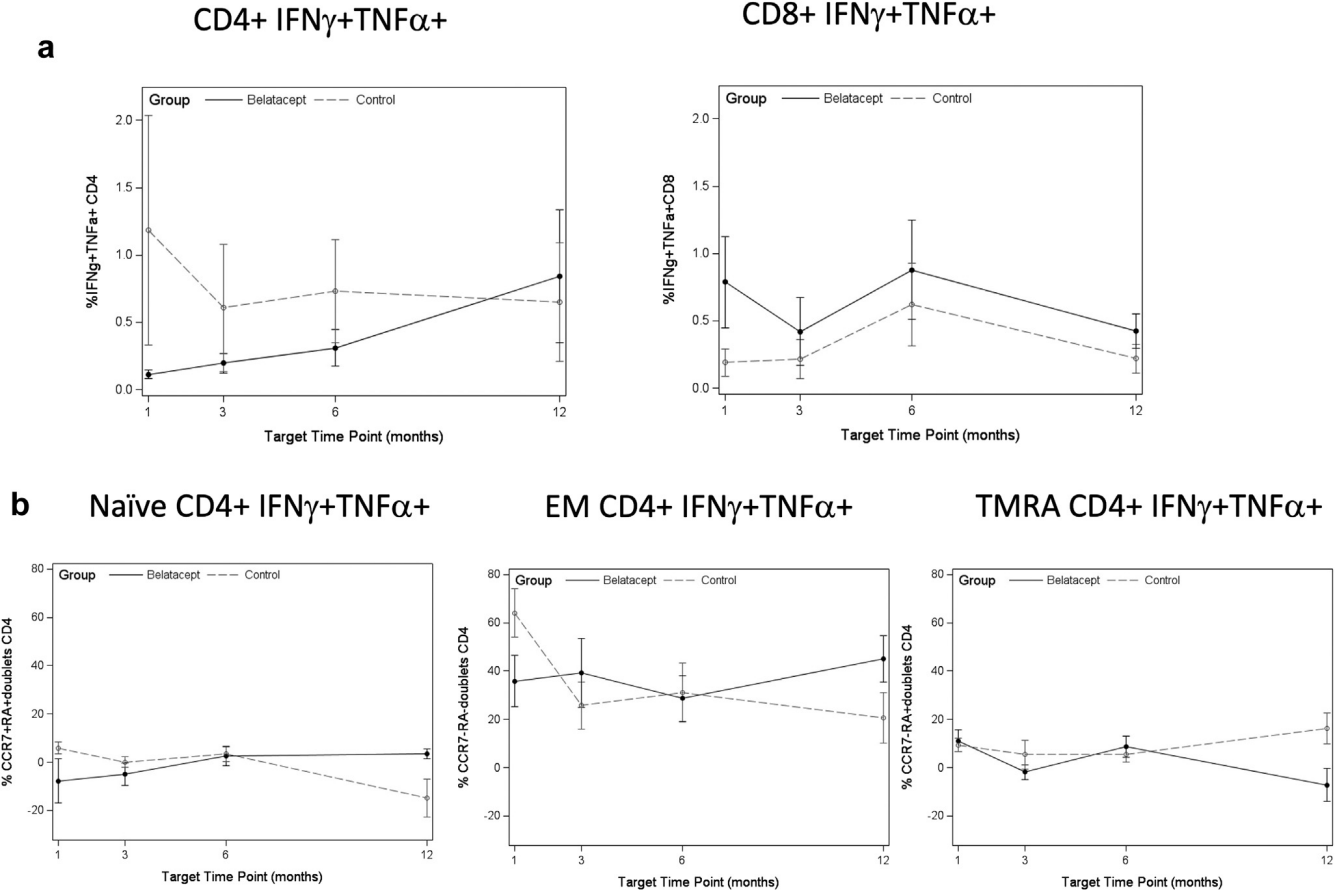

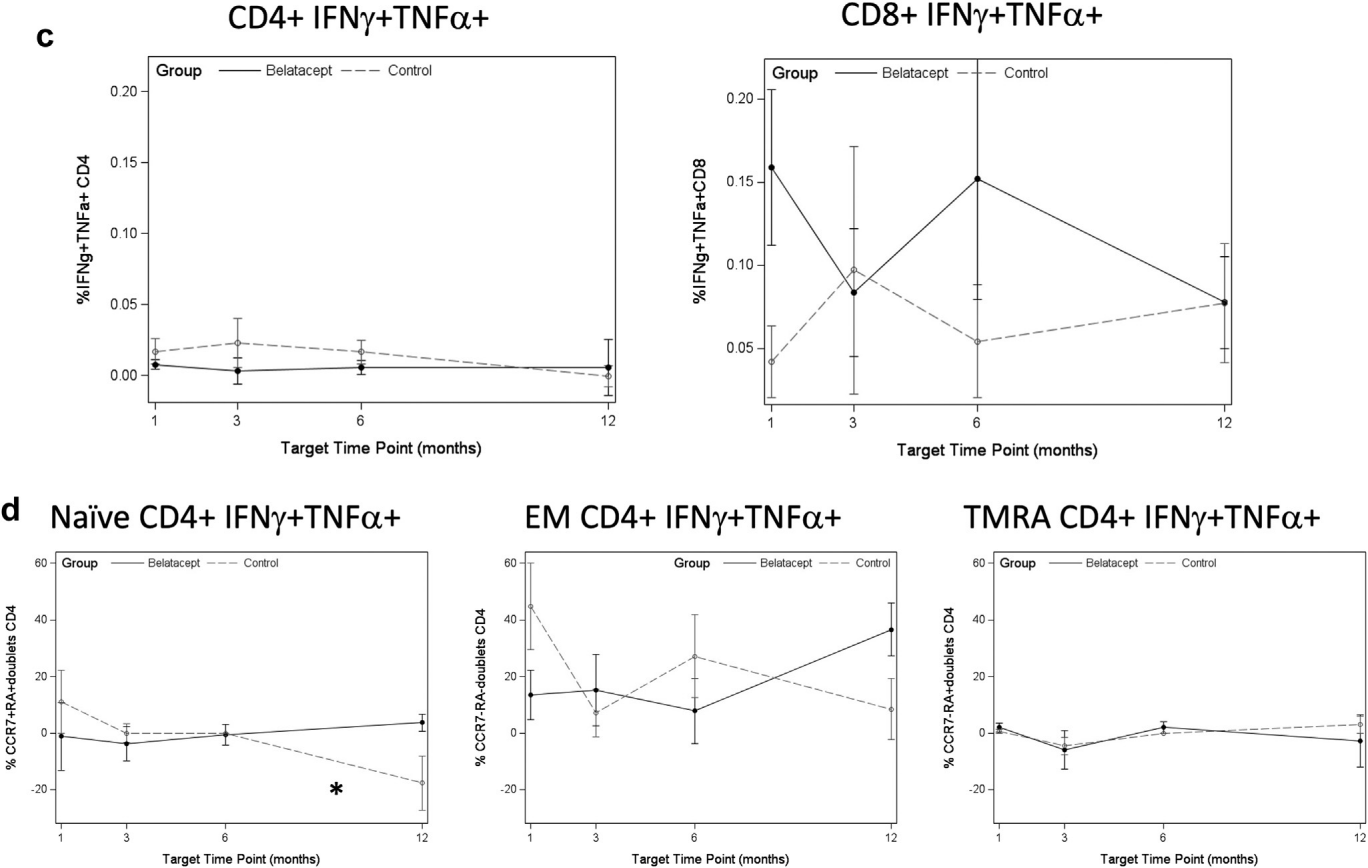
(a) Median frequency of IFN- γ/TNF-α double cytokine release from CMV-specific T cells at each time point after switch to belatacept, or equivalent time post-transplant for controls. Denominator is the total percentage of CD8 or CD4 T cells, as appropriate. (b) Median frequency of double cytokine release by maturation subtype from CMV-specific T cells at each time point after switch to belatacept, or equivalent time post-transplant for controls. (c) Median frequency of double cytokine release from EBV-specific T cells at each time point after switch to belatacept, or equivalent time post-transplant for controls. (d) Median frequency of double cytokine release by maturation subtype from EBV-specific T cells at each time point after switch to belatacept, or equivalent time post-transplant for controls. Belatacept patients indicated by solid points and solid lines; control patients indicated by gray points and dashed lines. For analysis by maturation subtype, denominator is the total percentage of IFN- γ/TNF-α CD8 or CD4 T cells, as appropriate. IFN-γ, interferon-γ; TNF-α, tumor necrosis factor-α.

**Figure 4:**
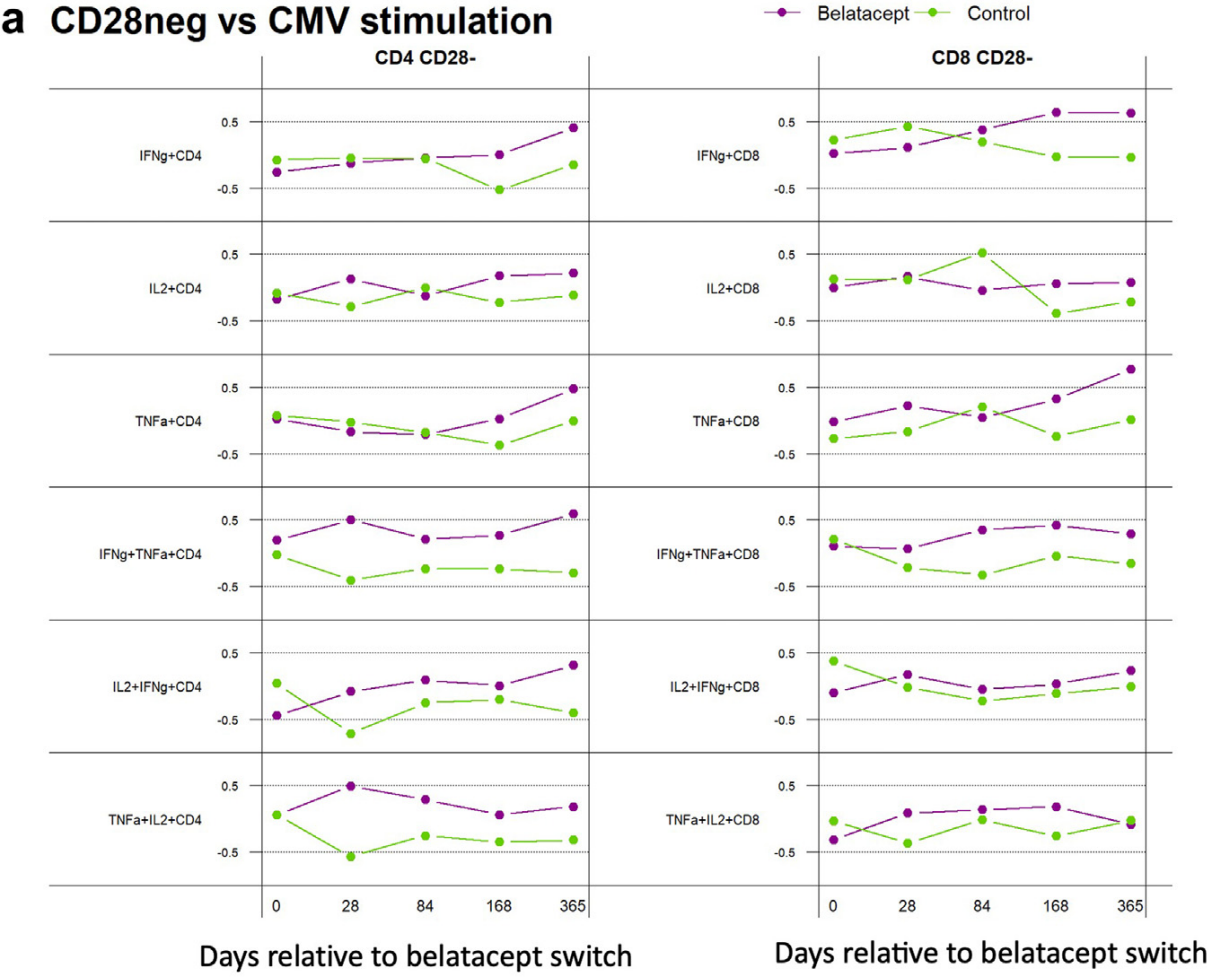

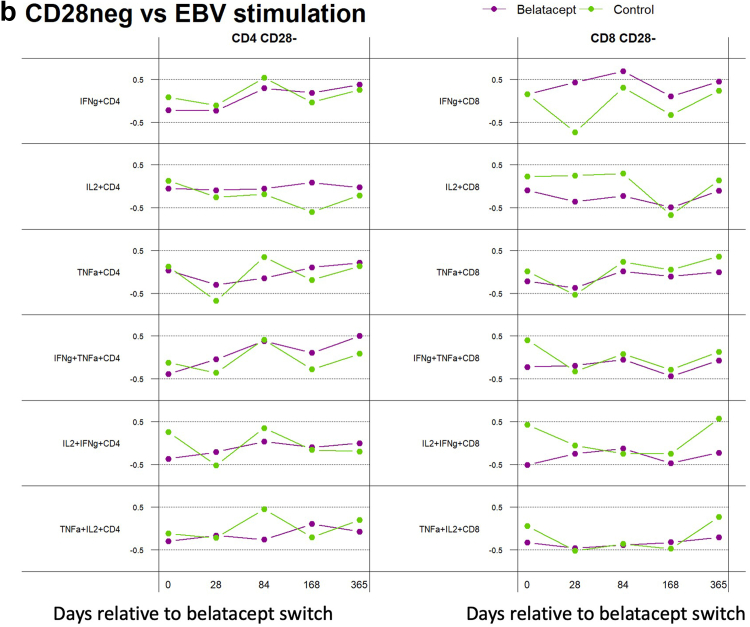
(a) Correlation between CD28− T-cell frequency and CMV immune response. For each subtype tested for response to CMV antigen, the degree of positive or negative correlation at each time point with CD28− T cells are shown for CD4 (left panel) and CD8 (right panel) CD28− T cells, Belatacept patients are shown in purple and control patients in green. (b) Correlation between CD28− T-cell frequency and CMV immune response. For each subtype tested for response to EBV antigen, the degree of positive or negative correlation at each time point with CD28− T cells are shown for CD4 (left panel) and CD8 (right panel) CD28− T cells, Belatacept patients are shown in purple and control patients in green. CMV, cytomegalovirus; EBV, Epstein Barr virus.

For comparing groups of interest, we first performed single-variable analysis with Mann-Whitney *U* test for continuous variables and Fisher exact test for categorical variables. Mixed-effects linear regression analysis was used to estimate the change from baseline over time within and between groups as shown in the Tables 1 and 2; and Figure 1, Figure 2, Figure 3. To address the issue of multiple comparisons and control for the false discovery rate (FDR), we calculated the FDR-adjusted *P* values. Statistical analysis was performed using SAS version 9.4 (SAS Institute Inc., Cary, NC). Additional analysis was performed to calculate the degree of correlation at each time point between CD28− T-cell frequency and CMV-specific or EBV-specific cytokine expression as shown in Figure 4 (R Core Team 2021, R Foundation for Statistical Computing).

## Results

### Clinical Outcomes

Of the 19 patients enrolled, demographic characteristics are shown in Table 1. No statistically significant differences were noted in rates of CMV viremia in the first year after transplantation. There were also no significant differences observed in terms of acute rejection between the belatacept or control group, although there was a trend toward increased frequency of DSA after transplant in the control group. There were no significant episodes of infection in either group. There were no cases of posttransplant lymphoproliferative disorder, and no patients died. One patient ended belatacept use after 6 months; the other 18 patients all extended on belatacept for at least 12 months.

**Table 1:** Demographic and clinical characteristics of patients switched to belatacept as compared with control patients maintained on standard immunosuppression

Patient characteristics	Belatacept (*n* = 19)	Control (*n* = 19)	*P* value
Median age (range)	57 (27–85)	52 (33–74)	0.397
Male	14 (73.7%)	15 (79.0%)	1.000
White race	4 (21.1%)	4 (21.1%)	1.000
Hispanic	7 (36.8%)	7 (36.8%)	1.000
Median PRA (range)	0 (0)	0 (0–83)	0.152
Baseline kidney disease	DM 8 (42%)	DM 5 (26%)	0.856
	HTN 2 (11%)	HTN 3 (16%)	
	GN 3 (16%)	GN 3 (16%)	
	Other 5 (26%)	Other 6 (11%)	
HLA mismatch (ABDRDQ) (range)	5 (3–8)	6 (1–7)	0.337
Cold ischemia time, h (range)	15 (1–26)	14 (1–23)	0.784
Induction, ATG	6 (31.6%)	6 (31.6%)	1.000
Deceased donor	8 (42.1%)	8 (42.1%)	1.000
Median time post-transplant to baseline (d) (IQR)	62 (49–90)	64 (42–85)	0.988
GFR at baseline (IQR)	33 (26–44)	51 (37–70)	0.002
Tacrolimus level at baseline (IQR)	8.9 (7.5–10.9)	9.5 (6.0–11.4)	0.950
CMV high risk (D+/R−)	3 (15.8%)	0 (0%)	0.230
CMV low risk (D−/R−)	2 (10.5%)	4 (21.1%)	
CMV seropositive	17 (89.4%)	12 (63.2%)	0.125
CMV viremia, first year	2 (10.5%)	7 (36.8%)	0.125
DSA, first year	0 (0%)	4 (21.1%)	0.105
Acute rejection, first year	1 (5.3%)	3 (15.8%)	0.604

ATG, antithymocyte globulin; CMV, cytomegalovirus; DSA, donor-specific antibody; GFR, glomerular filtration rate; HLA, human leukocyte antigen; IQR, interquartile range; PRA, panel-reactive antibody.

Control patients matched by age, living v deceased donor, and induction type, with available samples matched on time post-transplanted. Number (%) reported for categorical variables, and median (range or IQR) for continuous variables.

### Immune Phenotyping

Immune phenotype, including maturation subtype, Tregs, activation (HLADR+), immune senescence (CD28−, KLRG1+, CD57+), and exhaustion (PD-1+) for CD4+ and CD8+ T cells was measured at baseline and again at 3, 6, and 12 months after switch to belatacept, with corresponding time points measured at comparable times relative to transplant for controls. Baseline evaluation did not show significant differences between belatacept and control patients for any T-cell subtype (data not shown).

At baseline at the time of belatacept start, or cognate time point in controls, there were no significant differences observed in immune phenotype between the belatacept and control patients (data not shown). We analyzed change from baseline as slope over the first year after switch to belatacept. After transplant, there was a decrease in naive CD8+ T cells in patients switched to belatacept (*P* = 0.001, FDR *P* = 0.004) (Figure 1a), similar to control patients, who also demonstrated a decrease (Table 2). TMRA CD8+ and CD4+ T cells increased in patients switched to belatacept (*P* < 0.001, FDR *P* < 0.001 and *P* = 0.001, FDR *P* = 0.005, respectively), and these changes were not significantly different than control patients (Figure 1a and b). Increases were also observed for activated subsets CD4+ HLADR+, CD4+ CD57+, and CD8+ CD57+ T cells in all patients (Table 2 and Figure 1c and d).

**Table 2:** Analysis of slope of change from baseline in indicated immune phenotyping markers by treatment group in the 12 months after drug start or matched time after transplant for controls

Immune phenotype	Belatacept (*n* = 19), estimate (SE)	Belatacept *P* value	Control (*n* = 19), estimate (SE)	Control *P* value	Difference	Difference *P* value
CD8+ naive	−0.038 (0.010)	0.001^a^^,^^b^	−0.025 (0.011)	0.030^b^	−0.013 (0.015)	0.418
CD8+ CM	−0.005 (0.002)	0.066	−0.001 (0.002)	0.734	−0.004 (0.003)	0.304
CD8+ EM	−0.007 (0.008)	0.424	0.001 (0.000)	0.950	−0.007 (0.012)	0.553
CD8+ TMRA	0.050 (0.009)	<0.001^a^^,^^b^	0.026 (0.010)	0.016^b^	0.024 (0.014)	0.097
CD4+ naive	−0.006 (0.007)	0.429	−0.024 (0.008)	0.005^b^	0.018 (0.011)	0.125
CD4+ CM	0.000 (0.007)	0.951	0.012 (0.008)	0.125	−0.012 (0.011)	0.279
CD4+ EM	−0.006 (0.009)	0.518	0.005 (0.010)	0.656	−0.011 (0.014)	0.443
CD4+ TMRA	0.014 (0.004)	0.001^a^^,^^b^	0.006 (0.004)	0.175	0.007 (0.004)	0.184
CD8+ HLADR+	0.017 (0.012)	0.157	0.001 (0.012)	0.520	0.009 (0.017)	0.612
CD4+ HLADR+	0.019 (0.006)	0.002^a^^,^^b^	0.010 (0.006)	0.122	0.097 (0.000)	0.284
CD8+ CD57+	0.052 (0.012)	<0.001^a^^,^^b^	0.033 (0.013)	0.014^b^	0.019 (0.018)	0.305
CD4+ CD57+	0.031 (0.008)	0.004^a^^,^^b^	0.017 (0.008)	0.056	0.014 (0.012)	0.263
CD8+ CD28−	0.012 (0.006)	0.080	0.008 (0.007)	0.271	0.004 (0.010)	0.686
CD4+ CD28−	0.005 (0.010)	0.630	0.013 (0.11)	0.257	−0.008 (0.015)	0.621
CD8+ CD57+ CD28−	0.052 (0.012)	<0.001^a^^,^^b^	0.030 (0.013)	0.029^b^	0.022 (0.018)	0.239
CD4+ CD57+ CD28−	0.031 (0.008)	0.0003^a^^,^^b^	0.016 (0.008)	0.070	0.015 (0.010)	0.217
CD8+ CD57+ KLRG1+	0.023 (0.009)	0.015^b^	0.024 (0.009)	0.017^b^	−0.001 (0.010)	0.926
CD4+ CD57+ KLRG1+	0.018 (0.005)	0.001^a^	0.011 (0.005)	0.047^b^	0.006 (0.007)	0.402
CD8+ PD-1+	−0.008 (0.007)	0.311	0.068 (0.008)	0.422	−0.015 (0.011)	0.202
CD4+ PD-1+	0.022 (0.007)	0.004^a^	0.014 (0.007)	0.076	0.008 (0.010)	0.486
CD4+ Treg	−0.006 (0.004)	0.174	−0.001 (0.000)	0.768	−0.005 (0.006)	0.466

CM, central memory; EM, effector memory; SE, standard error; TMRA, terminally differentiated RA+ effector memory; Treg, T-regulatory cell.

Markers were measured as percentages, which are frequency of cell subtype of CD8+ or CD4+ T cells. Data are summarized as median (SE). False discovery rate–adjusted *P* value is indicated for each row.

a Comparisons with *P* < 0.05 by false discovery rate (FDR).

b Comparisons with unadjusted *P* < 0.05.

Increases were seen in senescent subtypes defined as CD57+CD28− and CD57+KLRG1+ in both CD8+ and CD4+ T cells patients switched from belatacept (Figure 2a and b), as well as in CD4+PD1+ T cells (Figure 2c). No significant difference was observed by comparison of slope for Tregs for either group (Table 2 and Figure 2d).

Repeating the analysis based on ATG compared with basiliximab induction regardless of belatacept versus control status also revealed differences between patient groups. Frequencies of naive CD8+ T cells decreased in both induction groups with a greater change after ATG in which a decrease of −0.050 (standard error [SE] 0.012) was seen compared with −0.018 (SE 0.007) for basiliximab) (*P* < 0.001 and *P* = 0.025, respectively), although the difference of −0.032 (SE 0.015) was not statistically significant with FDR correction (*P* = 0.235). TMRA CD8+ T cells increased in both induction groups, with an increase of 0.059 (SE 0.011) after ATG and 0.025 (SE 0.007) for basilixamab with a greater change after ATG (<0.001 for both), although the difference between groups of 0.033 (SE 0.010) was not significant with FDR correction (*P* = 0.134). A similar trend was seen for CD4+ T cells (data not shown). Senescent CD8+ T cells also increased over time in both groups with a trend toward greater change after ATG, although these differences did not reach statistical significance when corrected by FDR (data not shown).

### Alloreactivity

Donor-specific alloreactivity as assessed by ICS cytokine measurement was low to undetectable at study enrollment, and it did not change significantly in either belatacept or control patients overall. No significant difference was observed between belatacept and control patients at baseline. Mixed lymphocyte assessment using donor cells did not show significant differences by change in slope over time after switch to belatacept (Supplementary Table S1). This was true both for CD8+ and CD4+ T cells when analyzed either by single or double cytokine secretion for all combinations of IFN-γ, TNF-α, and interleukin-2 and when CD107+ or PD1+ T cells were analyzed (data not shown). Levels of alloreactivity were not associated with development of acute rejection for both belatacept and control patients (data not shown). No significant differences between patient groups were seen when third-party cells, the negative control, were used as stimulus instead of donor cells (data not shown).

Analysis of human leukocyte antigen class I and class II single-antigen testing revealed that 0 of 19 belatacept compared with 4 of 19 control patients demonstrated *de novo* DSA detected at one or more time points during the period of study (*P* = 0.035) (Supplementary Figure S2). For those with DSA detected after transplantation, mean fluorescence intensity ranged from 1030 to 7809, and the number of human leukocyte antigens recognized ranged from 1 to 5. Two patients had both class I and class II antibodies, whereas 1 patient had class I and 1 patient class II only. Analysis of association between detection of DSA and response to alloimmune stimulation demonstrated a significant increase in IFN-γ/TNF-α double cytokine secretion for CD8+ T cells for naive (difference of 0.201 [SE 0.099], *P* = 0.042) or effector memory subtypes (difference of 0.198 [SE 0.070], *P* = 0.005), although neither association was statistically significant after FDR correction. No significant association was seen between alloimmune response and development of rejection (data not shown). Interestingly, none of the patients with detectable DSA developed acute rejection, likely because the DSA detected was at a relatively low level (Supplementary Figure S2).

### CMV and EBV Antiviral Activity

At baseline, there were no significant differences observed in CMV or EBV antigen-specific immune response between the belatacept and control patients (data not shown). Antiviral immunity against CMV and EBV did not decrease after switch to belatacept: assessment of CMV-specific cytokine-secreting T cells did not show any significant decrease over the first year in patients switched to belatacept by change in slope (Supplementary Table S2). The slope of IFN-γ/TNF-α CD4+ T cells increased over the first year (*P* = 0.001) and was significantly different than control patients (*P* = 0.013), although this difference was not statistically significant when corrected by FDR (Figure 3a). When double-cytokine–secreting CD4+ T cells were subsetted by maturation subtypes, patients switched to belatacept had a decrease in TMRA cells specific to CMV (*P* = 0.014), and this was significantly different than control patients (*P* = 0.012), although not statistically significant when corrected by FDR. In contrast, control patients demonstrated a decrease in naive double-cytokine–secreting CD4+ T cells (*P* = 0.001), which was significantly different than patients switched to belatacept (*P* < 0.001), and these findings were significant by FDR (*P* = 0.044 and *P* = 0.021, respectively) (Figure 3b). These analyses did not differ when patients without CMV viremia were omitted from the analysis (data not shown).

Review of the median frequencies of CMV-specific response in IFN-γ/TNF-α CD8+ T cells by maturation subtype also revealed no significant differences (Supplementary Table S3). This demonstrates CMV-specific T-cell response, which was similar in both control and belatacept-switched patients.

Analysis of the EBV-specific immune response in patients switched to belatacept compared with controls was also performed. Single- and double-cytokine secretion was similar between belatacept and control patients (Supplementary Table S3) (Figure 3c). EBV-specific triple cytokine secretion from CD4+ T cells from control patients did exhibit a negative slope over the first-year after transplant (*P* < 0.001), which was significantly different compared with patients switched to belatacept (*P* = 0.001), with both of these observations significant by FDR-adjusted testing. When subsetted by maturation subtype, the naive CD4+ T cell IFN-γ/TNF-α double cytokine–secreting cells exhibited a negative slope in the control patients (*P* < 0.001), which was also significantly different compared with patients switched to belatacept (*P* = 0.001) and remained significant after FDR correction (Figure 3d). Overall, EBV-specific immune response was detectable and persistent in both patient groups.

As a follow-up analysis, we analyzed cytokine release after stimulation with Staphylococcus enterotoxin B to test non–antigen-specific cell function (Supplementary Table S4). Patients switched to belatacept had similar single cytokine secretion after Staphylococcus enterotoxin B stimulation, but a trend was seen toward increased frequency of triple cytokine–secreting CD4+ T cells, although this did not remain statistically significant after correction by FDR. However, when subsetted by maturation subtype, a significant difference was observed for naive CD4+T cells, which increased in belatacept patients but decreased in control patients, a difference which was statistically significant (*P* < 0.001), and this remained statistically significant after FDR correction (Supplementary Figure S2).

### Integrated Analysis of Immunophenotype and Antigen Response Therapy

To further analyze the relationship between immunophenotype, especially loss of the belatacept cofactor CD28, and functional antigen-specific response, we evaluated the correlation between frequency of CD28− T cells and IFN-γ/TNF-α and IL-2/TNF-α double cytokine release after CMV and EBV antigen stimulation (Figure 4). This revealed a differential association between patients switched to belatacept compared with control patients maintained on CNI: for patients on belatacept, the frequency of CD28− CD8+ T cells was positively correlated with the CMV-specific immune response, with a dynamic change over time demonstrating the strongest association 6 or 12 months after the switch, especially for IFN-γ and TNF-α single cytokine secretion (Figure 4a). A similar pattern was observed for CD28− CD4+ T cells, although the correlation was less pronounced, and the strongest association was observed for double cytokine IFN-γ/TNF-α–secreting cells. This correlation was not observed for control patients who remained on CNI during the comparative period. In contrast, the EBV-specific immune response showed less differential correlation between belatacept and control patients and CD28− CD8+ T-cell frequency (Figure 4b). Minimal correlation was observed between the CD28− CD4+ T-cell population and EBV-specific immune response in both patient groups. Given the very low levels of alloimmunity observed in this cohort, we were not able to repeat this integrated analysis using the alloimmune response.

## Discussion

We measured the immunologic impact of switch to belatacept in patients with intolerance to CNIs on immune phenotype, alloimmunity, and antiviral immunity over time using clinically relevant viruses CMV and EBV. This analysis uniquely examined the impact of the belatacept switch in comparison with a well-matched control cohort. Immune phenotyping demonstrated a stability of frequency of maturation subtypes, activation, senescence, and Tregs after the belatacept switch compared with control patients. This study therefore demonstrates a real-world approach to patient selection and timing and approach for switching to belatacept in conjunction with CNI taper, avoiding development of *de novo* DSA and rejection.

Another unique analysis from this cohort is the demonstration of an absence of alloimmune response in tandem with preservation of antiviral immune response in patients switched to belatacept. We hypothesize that this difference stems from the fact that alloimmune T cells in unsensitized patients are primarily naive CD4+ T cells that express CD28+, whereas antiviral T cells demonstrate lower levels of CD28+ expression, leading to relative freedom from belatacept inhibition. Our data suggest that these mature, virus-specific T cells are both CD4+ and CD8+, whereas double cytokine–secreting cells are CD4+ T cells. These observations parallel results from *in vitro* studies, with inhibition of alloimmune response but preservation of virus-specific immune response.^20^ However, an important distinction between our study and previous reports is the study of PBMCs collected from patients receiving belatacept in contrast to previous studies in which exogenous belatacept was added to PBMCs *in vitro* in healthy controls or kidney transplant patients receiving CNI.^19^^,^^20^

We have further extended our investigation through an integrated analysis of CD28− T-cell frequency and antiviral immune response, which revealed a positive correlation between CD28− T cells and CMV-specific single or double cytokine–producing cells for patients switched to belatacept (Figure 4). This suggests that memory T cells, experienced cells often with senescent features such as loss of CD28, are relatively immune to the impact of belatacept, whereas the naive CD28+ T cells are more likely to be responsible for allo-antigen immune response and are suppressed with belatacept treatment. Interestingly, for the patients on belatacept, the frequency of these cells was observed to increase over time especially for CMV, suggesting the possibility of the so-called escape mechanism occurring for the antiviral immune response shifting to a predominately CD28-negative phenotype as previously described for alloreactive T cells.^26^

Both control and belatacept patients demonstrated the impact of immunosuppression with a decrease in naive T cells and increase in TMRA T cells (Table 2). This finding is notable given the reported association between increased frequency of effector memory or TMRA memory cells and rejection resistant to belatacept treatment in human transplant recipients as well as in nonhuman primate models.^27^, ^28^, ^29^ TMRA cells may be associated with rates of rejection and rejection resistant to belatacept therapy.^30^ Another intriguing finding was the increase in activated T cells by HLADR+ or CD57+ expression regardless of induction type, seen in both belatacept and control patients, a finding found to be associated with belatacept-resistant rejection in human subjects.^31^ Monitoring of the frequency of immune subtypes associated with protection against rejection has promise for selecting patients likely to benefit from belatacept therapy.^32^^,^^33^

Patients switched to belatacept maintained stable levels of alloreactivity as defined by mixed lymphocyte reaction with donor cells (Supplementary Table S1). This was associated with a relative freedom from rejection. Interestingly, control patients demonstrated a decrease in naive and effector memory double-cytokine–secreting alloreactive CD4 T cells, associated with development of DSA. This suggests that the absence of an increase in the alloreactive T-cell response may be the mechanism behind beneficial effect of belatacept on rejection, through costimulation blockade preventing development of the memory T-cell response to *de novo* alloantigens.^34^^,^^35^

In analysis of the antiviral immune response, no significant impact was found on CMV or EBV-specific immune response in patients switched to belatacept. In fact, control patients demonstrated a decrease in naive double-cytokine CMV-specific T cells, suggesting possible impaired antiviral immune response, whereas belatacept-switched patients demonstrated no significant decrease in CMV- or EBV-specific T-cell response (Supplementary Tables S3 and S4), with memory cells likely providing protection against clinically CMV infection.^36^ These findings are in contrast to other reports of impaired CMV immune response,^17^ potentially because we used peptides representing multiple CMV antigens for stimulation and measured the expression of multiple cytokines simultaneously, allowing for a more complete evaluation of antiviral response. The observation of preserved antiviral function in the face of impaired alloimmunity could reflect the fact that TCR avidity toward viral antigens is generally higher than alloantigens, requiring less costimulation and therefore more difficult to inhibit with costimulation-inhibitor.^37^ This may explain the low incidence of CMV DNAemia and disease in our cohort, which was predominantly CMV R+, in contrast to reports of increased CMV disease in CMV D+R− kidney transplant recipients.^16^^,^^38^ Of note, EBV antiviral response was better preserved in patients receiving belatacept compared with CNI therapy, possibly because of the fact that EBV is typically a memory response in adults and therefore less affected by second signal inhibition. Analysis of T-cell activity by Staphylococcus enterotoxin B response found no significant difference after switch to belatacept as compared with control patients. This preservation of immune function of patients on belatacept compared with control was most noticeable in the naive double cytokine–secreting CD4 T-cell subset, possibly reflecting the differential impact of CNI on CD4 compared with CD8 T cells.^39^ These observations demonstrate that despite absence of alloreactive immune response in patients switched to belatacept, these patients maintain ability to response to microbial pathogens.

A limitation of this study is the relatively small cohort size, although it is similar in size to other mechanistic studies of the allograft immune response. This limitation is mitigated by the precise matching of belatacept to control patients, the homogeneity of clinical care provided by a single-center study following protocolized care for infection and rejection prevention, and the fact that the timing of immunologic analysis relative to transplantation was matched in the belatacept and control arms. Future studies can include a more diverse group in terms of pretransplant sensitization and rejection risk and will assess T-follicular helper cells, which influence B-cell differentiation and play an important role in the development of DSA.^30^^,^^40^ However, previous data suggest that even in sensitized patients, belatacept remains effective for prevention of DSA persistence and that DSA that do emerge are of lower mean fluorescence intensity compared with CNI controls.^41^, ^42^, ^43^, ^44^ A larger cohort will also allow for investigation of the specific impact of induction immunosuppression, the role of CD28-negative antigen-specific immune response, and the impact on cellular proliferation on patients receiving belatacept versus conventional therapy.

Our studies revealed that after switch to belatacept, immune phenotype, alloreactivity, and antiviral activity remain overall similar over time in patients switched to belatacept compared with control patients on CNI therapy, suggesting that patients with or without CNI toxicity evidence may benefit from the belatacept switch. Management of concurrent immunosuppression as in our study via continuation of prednisone, or replacement of mycophenolate mofetil with a mechanistic target of rapamycin inhibitor, may be important approaches for avoiding rejection with belatacept use.^45^ Therefore, this real-world evaluation of stability of immune phenotype, absence of alloimmune response, and preservation of antiviral immune response supports the use of belatacept for maintenance immunosuppression in low-risk patients unable to tolerate the conventional CNI regimen.

## Disclosure

Support for this study was provided by an investigator-initiated study grant from Bristol Myers Squib (principal investigator, Bunnapradist). ER, SB, and JS also received support from the National Institutes of Health (grant U19-AI-128913-01). All the other authors declared no competing interests.

## Supplementary Material

Supplementary File (PDF)

**Figure S1.** Dot plot of representative flow cytometry experiment.

**Figure S2.** Donor-specific antibodies by MFI over time for the 4 control patients with detectable HLA Class I or II antibodies after transplantation.

**Figure S3.** Median frequency of double cytokine release by maturation subtype from SEB-stimulated T cells at each time point after switch to belatacept, or equivalent time post-transplant for controls.

**Table S1.** Analysis of slope of change from baseline in indicated intracellular cytokine staining results by treatment group, with stimulation by mixed lymphocyte reaction using donor cells for stimulation.

**Table S2.** Analysis of slope of change from baseline in indicated intracellular cytokine staining results by treatment group, with stimulation by CMV antigen.

**Table S3.** Analysis of slope of change from baseline in indicated intracellular cytokine staining results by treatment group, with stimulation by EBV antigen.

**Table S4.** Analysis of slope of change from baseline in indicated intracellular cytokine staining results by treatment group, with stimulation by Staphylococcal endotoxin B (SEB) for stimulation.

**STROBE/CONSORT Checklist** (Word).
