# Supplementary material for: Preservation of Antiviral Immunologic Efficacy Without Alloimmunity After Switch to Belatacept in Calcineurin Inhibitor–Intolerant Patients
Source: Kidney Int Rep. 2022 Oct 20;8(1):126–40. doi: 10.1016/j.ekir.2022.10.015 (PMC9832066; doi:10.1016/j.ekir.2022.10.015)
Supplement: Supplementary File (PDF) [file mmc1.pdf]

Supplementary Tables and Figures

Supplementary Table 1

Analysis of slope of change from baseline in indicated intracellular cytokine staining results by treatment group, with stimulation by mixed lymphocyte reaction using donor cells for stimulation. Time period of analysis is the 12 months after drug start, or matched time post-transplant for controls. Markers were measured as percentages, which are frequency of cell subtype out of CD8+ or CD4+ T cells. Data were summarized as Median (SE). FDR-adjusted p-value is indicated for each row. Abbreviations: CM, central memory; EM, effector memory; TMRA, terminally differentiated RA+ effector memory. Bold indicates comparisons with p-value <0.05. Asterisk indicates p-value <0.05 by FDR.

Immune phenotype	Belatacept (n=19) Estimate (SE)	p-value Belatacept	Control (n=19) Estimate (SE)	p-value Control	Difference (SE)	p-value Difference
**CD8+ IFN-γ**	-0.000 (0.011)	0.986	0.001 (0.013)	0.924	-0.001 (0.017)	0.933
**CD8+ TNF-α**	0.000 (0.009)	0.956	0.004 (0.010)	0.680	-0.003 (0.013)	0.784
**CD8+ IL2+**	0.001 (0.006)	0.806	0.003 (0.006)	0.645	-0.001 (0.009)	0.852
**CD8+ IFN-γ/TNF-α**	-0.000 (0.001)	0.962	0.000 (0.001)	0.905	-0.000 (0.002)	0.596
**CD8+ TNF-α/IL2+**	-0.000 (0.000)	0.933	0.00 (0.000)	0.787	-0.00 (0.000)	0.795
**CD8+ IFN-γ/IL2+**	-0.000 (0.000)	0.992	0.000 (0.000)	0.926	-0.000 (0.000)	0.934
**CD8+ IFN-γ/TNF-α/IL2+**	0.037 (0.025)	0.139	0.016 (0.029)	0.576	0.021 (0.038)	0.581

**CD4+ IFN-γ**	-0.000 (0.039)	0.992	0.001 (0.044)	0.989	-0.001 (0.059)	0.987
**CD4+ TNF-α**	0.001 (0.020)	0.950	0.004 (0.023)	0.856	-0.003 (0.030)	0.924
**CD4+ IL2+**	0.014 (0.015)	0.347	0.002 (0.017)	0.908	0.012 (0.022)	0.596
**CD4+ IFN-γ/TNF-α**	-0.000 (0.001)	0.950	0.000 (0.001)	0.597	-0.001 (0.001)	0.660
**CD4+ TNF-α/IL2+**	-0.000 (0.001)	0.897	0.000 (0.001)	0.885	-0.000 (0.002)	0.846
**CD4+ IFN-γ/IL2+**	-0.000 (0.001)	0.863	0.000 (0.001)	0.899	-0.000 (0.001)	0.834
**CD4+ IFN-γ/TNF-α/IL2+**	-0.024 (0.039)	0.536	-0.102 (0.045)	**0.025**	0.077 (0.060)	0.196
**IFN-γ/TNF-α CD8+ naïve**	0.002 (0.033)	0.953	0.018 (0.038)	0.633	-0.016 (0.051)	0.748
**IFN-γ/TNF-α CD8+ CM**	0.003 (0.022)	0.892	0.030 (0.025)	0.233	-0.018 (0.039)	0.640
**IFN-γ/TNF-α CD8+ EM**	0.008 (0.024)	0.733	0.049 (0.27)	0.077	-0.040 (0.036)	0.266
**IFN-γ/TNF-α CD8+ TMRA**	-0.016 (0.028)	0.565	0.009 (0.03)	0.781	-0.025 (0.042)	0.557
**IFN-γ/TNF-α CD4+ naive**	0.039 (0.024)	0.096	-0.107 (0.027)	**<0.001***	0.146 (0.036)	**<0.001***
**IFN-γ/TNF-α CD4+ CM**	-0.005 (0.026)	0.839	0.013 (0.029)	0.657	-0.018 (0.039)	0.638
**IFN-γ/TNF-α CD4+EM**	0.002 (0.035)	0.950	-0.084 (0.041)	**0.037**	0.087 (0.054)	0.106
**IFN-γ/TNF-α CD4+ TMRA**	-0.014 (0.020)	0.491	0.003 (0.023)	0.884	-0.017 (0.031)	0.574

Supplementary Table 2

Analysis of slope of change from baseline in indicated intracellular cytokine staining results by treatment group, with stimulation by CMV antigen. Time period of analysis is the 12 months after drug start, or matched time post-transplant for controls. Markers were measured as percentages, which are frequency of cell subtype out of CD8+ or CD4+ T cells. Data were summarized as Median (SE). FDR-adjusted p-value is indicated for each row. Abbreviations: CM, central memory; EM, effector memory; TMRA, terminally differentiated RA+ effector memory. Bold indicates comparisons with p-value <0.05. Asterisks indicate p-value <0.05 by FDR. 6 patients excluded where both donor and recipient were CMV seronegative.

Immune phenotype	Belatacept (n=17) Estimate (SE)	p-value Belatacept	Control (n=15) Estimate (SE)	p-value Control	Difference (SE)	p-value Difference
**CD8+ IFN-γ**	0.002 (0.012)	0.899	-0.005 (0.015)	0.761	0.006 (0.015)	0.753
**CD8+ TNF-α**	0.001 (0.009)	0.955	-0.002 (0.011)	0.850	0.003 (0.014)	0.855
**CD8+ IL2+**	-0.001 (0.006)	0.902	0.001 (0.007)	0.920	-0.001 (0.010)	0.876
**CD8+ IFN-γ/TNF-α**	-0.001 (0.001)	0.493	0.000 (0.002)	0.941	-0.001 (0.002)	0.624
**CD8+ TNF-α/IL2+**	-0.000 (0.000)	0.874	0.000 (0.000)	0.995	-0.000 (0.000)	0.916
**CD8+ IFN-γ/IL2+**	-0.000 (0.000)	0.797	0.000 (0.000)	0.994	-0.000 (0.001)	0.875
**CD8+ IFN-γ/TNF-α/IL2+**	0.024 (0.025)	0.344	0.018 (0.031)	0.562	0.055 (0.040)	0.890
**CD4+ IFN-γ**	0.004 (0.042)	0. 924	0.000 (0.050	0. 999	0.004 (0.065	0. 952

**CD4+ TNF-α**	0.007 (0.021)	0.770	0.005 (0.026)	0.854	0.001 (0.033)	0.966
**CD4+ IL2+**	0.007 (0.015)	0.678	0.002 (0.019)	0.920	0.005 (0.025)	0.854
**CD4+ IFN-γ/TNF-α**	0.003 (0.001)	**0.001**	-0.000 (0.001)	0.781	0.003 (0.001)	**0.022**
**CD4+ TNF-α/IL2+**	0.000 (0.001)	0.997	0.002 (0.001)	0.232	-0.002 (0.002)	0.355
**CD4+ IFN-γ/IL2+**	-0.000 (0.001)	0.841	0.002 (0.001)	**0.022**	-0.002 (0.001)	0.056
**CD8+ IFN-γ/TNF-α/IL2+**	-0.035 (0.040)	0.383	-0.105 (0.049)	**0.031**	0.071 (0.063)	0.260
**IFN-γ/TNF-α CD8+ naïve**	0.014 (0.035)	0.685	-0.026 (0.043)	0.539	0.041 (0.0555)	0.463
**IFN-γ/TNF-α CD8+ CM**	0.010 (0.023)	0. 609	-0.031 (0.028)	0. 271	0.043 (0.037)	0. 239
**IFN-γ/TNF-α CD8+ EM**	-0.002 (0.024)	0.941	0.017 (0.030)	0.566	-0.019 (0.038)	0.623
**IFN-γ/TNF-α CD8+ TMRA**	0.016 (0.028)	0.574	0.057 (0.034)	0.097	-0.041 (0.044)	0.350
**IFN-γ/TNF-α CD4+ naive**	0.039 (0.025)	0.115	-0.107 (0.031)	**<0.001***	0.147 (0.040)	**<0.001***
**IFN-γ/TNF-α CD4+ CM**	0.051 (0.027)	0.058	-0.002 (0.033)	0.947	0.053 (0.042)	0.211
**IFN-γ/TNF-α CD4+EM**	-0.019 (0.035)	0.592	-0.077 (0.043)	0.077	0.058 (0.056)	0.084
**IFN-γ/TNF-α CD4+ TMRA**	-0.052 (0.021)	**0.016**	0.036 (0.026)	0.178	-0.087 (0.034)	**0.010**

Supplementary Table 3

Analysis of slope of change from baseline in indicated intracellular cytokine staining results by treatment group, with stimulation by EBV antigen. Time period of analysis is the 12 months after drug start, or matched time post-transplant for controls. Markers were measured as percentages, which are frequency of cell subtype out of CD8+ or CD4+ T cells. Data were summarized as Median (SE). FDR-adjusted p-value is indicated for each row. Abbreviations: CM, central memory; EM, effector memory; TMRA, terminally differentiated RA+ effector memory. Bold indicates comparisons with p-value <0.05. Asterisk indicate p-value <0.05 by FDR. All subjects were EBV seropositive.

Immune phenotype	Belatacept (n=19) Estimate (SE)	p-value Belatacept	Control (n=19) Estimate (SE)	p-value Control	Difference (SE)	p-value Difference
**CD8+ IFN-γ**	-0.000 (0.011)	0.981	0.003 (0.014)	0.810	-0.003 (0.017)	0.849
**CD8+ TNF-α**	0.001 (0.009)	0.891	0.005 (0.010)	0.647	--0.004 (0.013)	0.794
**CD8+ IL2+**	0.001 (0.006)	0.845	0.001 (0.007)	0.936	-0.001 (0.009)	0.948
**CD8+ IFN-γ/TNF-α**	-0.000 (0.001)	0.861	0.000 (0.001)	0.794	-0.001 (0.002)	0.755
**CD8+ TNF-α/IL2+**	-0.000 (0.000)	0.834	0.00 (0.000)	0.834	-0.00 (0.000)	0.659
**CD8+ IFN-γ/IL2+**	-0.000 (0.000)	0.850	0.000 (0.000)	0.825	-0.000 (0.000)	0.771
**CD8+ IFN-γ/TNF-α/IL2+**	0.003 (0.025)	0.893	-0.015 (0.029)	0.618	0.018 (0.039)	0.640
**CD4+ IFN-γ**	-0.000 (0.039)	0.994	0.003 (0.045)	0.940	-0.004 (0.060)	0.951
**CD4+ TNF-α**	0.001 (0.020)	0.979	0.005 (0.023)	0.832	-0.004 (0.030)	0.885

**CD4+ IL2+**	0.003 (0.015)	0.812	0.001 (0.017)	0.952	0.002 (0.023)	0.917
**CD4+ IFN-γ/TNF-α**	-0.000 (0.001)	0.987	0.001 (0.001)	0.499	-0.001 (0.001)	0.599
**CD4+ TNF-α/IL2+**	-0.000 (0.001)	0.862	0.000 (0.001)	0.815	-0.001 (0.002)	0.771
**CD4+ IFN-γ/IL2+**	-0.000 (0.001)	0.871	0.000 (0.001)	0.809	-0.000 (0.001)	0.773
**CD4+ IFN-γ/TNF-α/IL2+**	0.005 (0.039)	0.889	-0.201 (0.047)	**<0.001***	0.207 (0.061)	**0.001***
**IFN-γ/TNF-α CD8+ naïve**	-0.004 (0.033)	0.896	-0.020 (0.040)	0.607	0.016 (0.052)	0.758
**IFN-γ/TNF-α CD8+ CM**	0.007 (0.022)	0.753	-0.024 (0.026)	0.341	0.031 (0.039)	0.352
**IFN-γ/TNF-α CD8+ EM**	0.016 (0.024)	0.510	0.033 (0.28)	0.244	-0.017 (0.037)	0.642
**IFN-γ/TNF-α CD8+ TMRA**	-0.002 (0.028)	0.933	0.047 (0.033)	0.156	-0.049 (0.043)	0.255
**IFN-γ/TNF-α CD4+ naive**	0.022 (0.024)	0.363	-0.115 (0.028)	**<0.001***	0.136 (0.036)	**<0.001***
**IFN-γ/TNF-α CD4+ CM**	-0.000 (0.026)	0.999	-0.048 (0.030)	0.110	0.048 (0.040)	0.223
**IFN-γ/TNF-α CD4+EM**	0.041 (0.035)	0.250	-0.081 (0.042)	0.052	0.122 (0.055)	**0.026**
**IFN-γ/TNF-α CD4+ TMRA**	-0.019 (0.020)	0.354	0.016 (0.024)	0.488	-0.035 (0.031)	0.259

Supplementary Table 4

Analysis of slope of change from baseline in indicated intracellular cytokine staining results by treatment group, with stimulation by Staphylococcal endotoxin B (SEB) for stimulation. Time period of analysis is the 12 months after drug start, or matched time post-transplant for controls. Markers were measured as percentages, which are frequency of cell subtype out of CD3+, CD8+ or CD4+ T cells. Data were summarized as Median (SE). FDR-adjusted p-value is indicated for each row. Abbreviations: CM, central memory; EM, effector memory; TMRA, terminally differentiated RA+ effector memory. Bold indicates comparisons with p-value <0.05. Asterisk indicates p-value <0.05 by FDR.

Immune phenotype	Belatacept (n=19) Estimate (SE)	p-value Belatacept	Control (n=19) Estimate (SE)	p-value Control	Difference (SE)	p-value Difference
**CD8+ IFN-γ**	0.011 (0.012)	0.325	-0.018 (0.014)	0.203	0.029 (0.018)	0.108
**CD8+ TNF-α**	0.009 (0.009)	0.303	-0.026 (0.010)	**0.014**	0.035 (0.014)	**0.011**
**CD8+ IL2+**	0.003 (0.006)	0.600	-0.008 (0.007)	0.239	0.011 (0.009)	0.215
**CD8+ IFN-γ/TNF-α**	0.005 (0.001)	**<0.001***	-0.001 (0.001)	0.608	0.005 (0.002)	**0.005**
**CD8+ TNF-α/IL2+**	0.000 (0.000)	0.138	-0.000 (0.000)	0.234	-0.00 (0.000)	0.967
**CD8+ IFN-γ/IL2+**	-0.001 (0.000)	0.075	0.000 (0.000)	0.429	-0.000 (0.000)	0.588
**CD8+ IFN-γ/TNF-α/IL2+**	0.004 (0.025)	0.860	0.007 (0.030)	0.814	-0.002 (0.039)	0.947
**CD4+ IFN-γ**	0.070 (0.039)	0.071	0.013 (0.046)	0.771	0.058 (0.060)	0.342

**CD4+ TNF-α**	0.066 (0.020)	**0.001**	0.010 (0.023)	0.676	0.056 (0.031)	0.073
**CD4+ IL2+**	0.022 (0.015)	0.133	-0.000 (0.018)	0.991	0.023 (0.023)	0.331
**CD4+ IFN-γ/TNF-α**	0.003 (0.001)	**<0.001***	-0.001 (0.001)	0.132	0.005 (0.001)	**0.001***
**CD4+ TNF-α/IL2+**	0.003 (0.001)	**0.004**	-0.002 (0.001)	0.127	0.005 (0.002)	**0.003**
**CD4+ IFN-γ/IL2+**	0.001 (0.001)	**0.035**	-0.000 (0.001)	0.520	0.002 (0.001)	0.064
**CD4+ IFN-γ/TNF-α/IL2+**	0.005 (0.040)	0.892	-0.106 (0.047)	**0.025**	0.112 (0.062)	**0.071**
**IFN-γ/TNF-α CD8+ naïve**	0.030 (0.034)	0.374	-0.006 (0.040)	0.874	0.036 (0.053)	0.488
**IFN-γ/TNF-α CD8+ CM**	-0.007 (0.022)	0.746	0.008 (0.026)	0.768	-0.015 (0.034)	0.664
**IFN-γ/TNF-α CD8+ EM**	-0.022 (0.024)	0.353	0.007 (0.029)	0.805	-0.029 (0.038)	0.432
**IFN-γ/TNF-α CD8+ TMRA**	-0.037 (0.028)	0.184	0.038 (0.033)	0.250	-0.076 (0.044)	0.083
**IFN-γ/TNF-α CD4+ naive**	0.046 (0.024)	0.054	-0.103 (0.028)	**<0.001***	0.149 (0.037)	**<0.001***
**IFN-γ/TNF-α CD4+ CM**	0.038 (0.026)	0.146	0.001 (0.031)	0.987	0.037 (0.040)	0.357
**IFN-γ/TNF-α CD4+EM**	-0.017 (0.036)	0.627	-0.054 (0.043)	0.203	0.037 (0.055)	0.506
**IFN-γ/TNF-α CD4+ TMRA**	-0.024 (0.020)	0.243	0.004 (0.024)	0.869	-0.027 (0.032)	0.380

Supplemental Figure 1

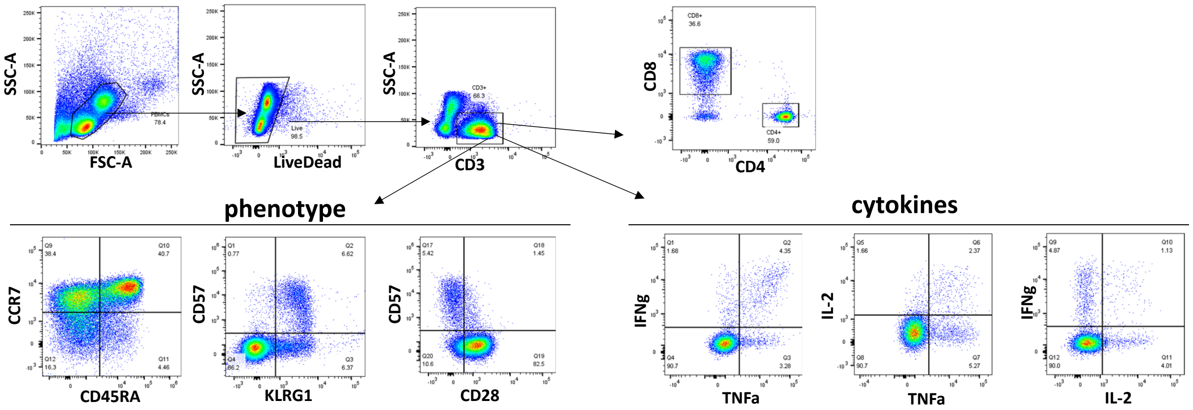

Supplemental Figure 1: Dot plot of representative flow cytometry experiment. Thawed PBMC were initially analyzed by side scatter (SSC-A) and forward scatter (FSC-A) to identify cells, followed by LiveDead gating to identify viable cells. CD3 was used to identify T lymphocytes, followed by separation by CD4 or CD8 expression. For immune phenotype identification, maturation subtypes were identified using CD45RA and CCR7, while CD57, KLRG1, and CD28 were used to identify senescent and exhausted cells. For antigen-specific testing, intracellular cytokine staining was performed after permeabilization for IFN-γ, TNF-α, and IL-2.

Supplemental Figure 2

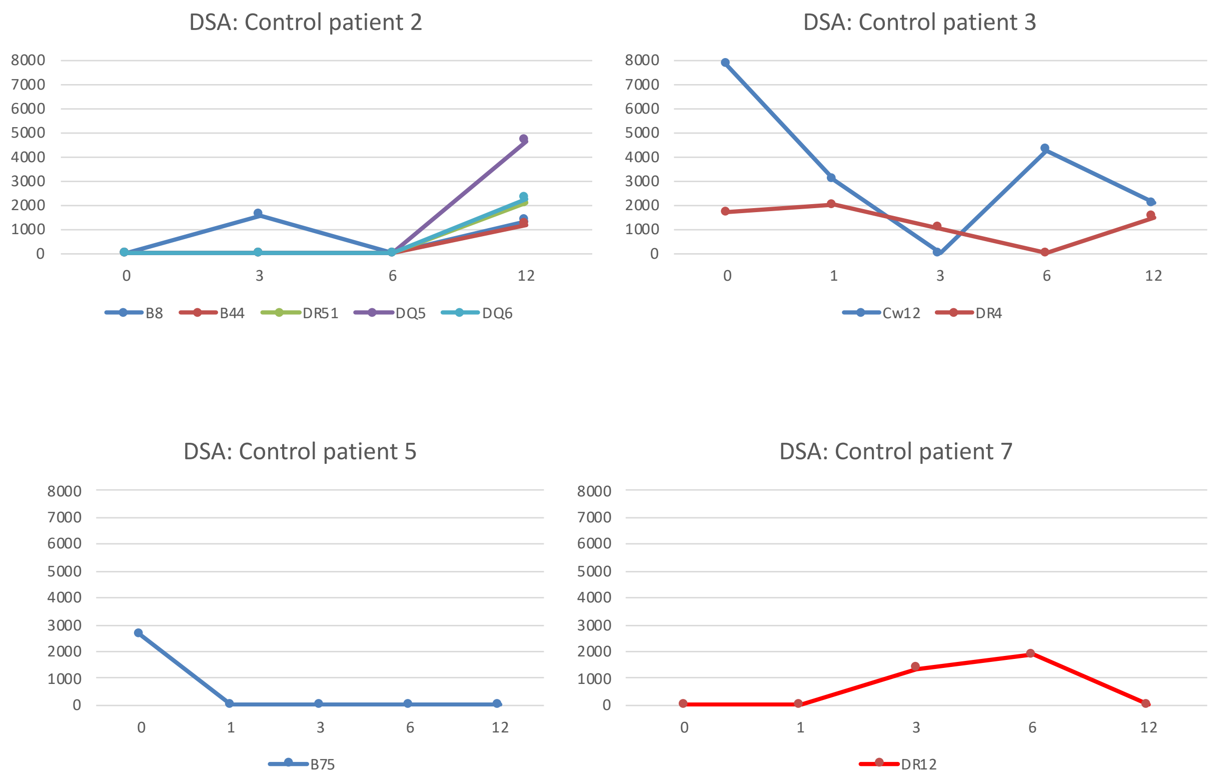

Supplemental Figure 2: Donor specific antibodies (DSA) by MFI over time for the four control patients with detectable HLA Class I or II antibodies after transplantation. Baseline represents a timepoint equivalent to switch to belatacept for each matched control patient. Each antigen is indicated in a different color. No belatacept patients developed detectable HLA Class I or II antibodies after transplantation. DSA testing was performed at all study timepoints and was negative for all subjects not shown in this Figure.

Supplemental Figure 3

SEB

Naïve CD4+ IFNγ+TNFα+ EM CD4+ IFNγ+TNFα+ TMRA CD4+ IFNγ+TNFα+

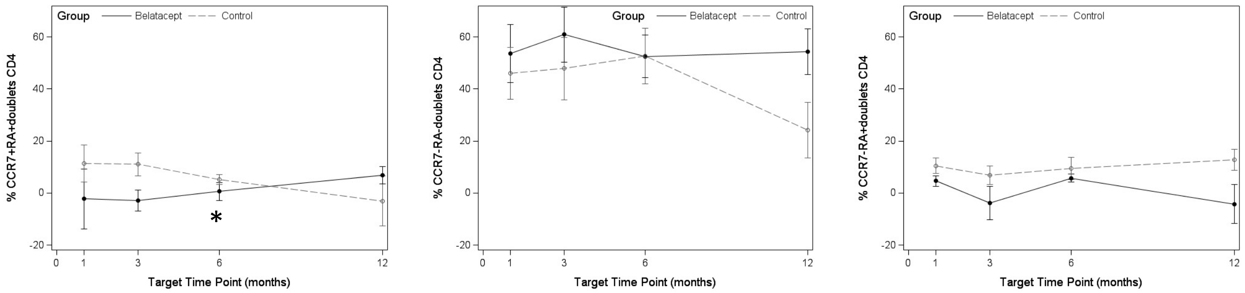

Supplemental Figure 3: Median frequency of double cytokine release by maturation subtype from SEB-stimulated T cells at each time point after switch to belatacept, or equivalent time post-transplant for controls. Belatacept patients indicated by solid points and solid lines; control patients indicated by grey points and dashed lines. Denominator is the total percentage of IFNγ/TNFα CD8 or CD4 T cells, as appropriate.
